# Reassortment Networks and the Evolution of Pandemic H1N1 Swine-Origin Influenza

**DOI:** 10.1109/TCBB.2011.95

**Published:** 2011-06-06

**Authors:** Shahid H. Bokhari, Laura W. Pomeroy, Daniel A. Janies

**Affiliations:** Department of Biomedical InformaticsOhio State University 333 W 10th Ave. Columbus Ohio 43210

**Keywords:** Cray XMT, graph theory, influenza, multithreading, networks, pandemic, reassortment, shortest paths, S-OIV, swine flu

## Abstract

Prior research developed *Reassortment Networks* to reconstruct the evolution of segmented viruses under both reassortment and mutation. We report their application to the swine-origin pandemic H1N1 virus (S-OIV). A database of all influenza A viruses, for which complete genome sequences were available in Genbank by October 2009, was created and dynamic programming was used to compute distances between all corresponding segments. A reassortment network was created to obtain the minimum cost evolutionary paths from all viruses to the exemplar S-OIV A/California/04/2009. This analysis took 35 hours on the Cray Extreme Multithreading (XMT) supercomputer, which has special hardware to permit efficient parallelization. Six specific H1N1/H1N2 *bottleneck* viruses were identified that almost always lie on minimum cost paths to S-OIV. We conjecture that these viruses are crucial to S-OIV evolution and worthy of careful study from a molecular biology viewpoint. In phylogenetics, ancestors are typically medians that have no functional constraints. In our method, ancestors are not inferred, but rather chosen from previously observed viruses along a path of mutation and reassortment leading to the target virus. This specificity and functional constraint render our results actionable for further experiments *in vitro* and *in vivo*.

## 1 Introduction

A newly emergent strain of influenza subtype H1N1, termed the swine origin influenza virus (S-OIV), was first detected during an outbreak in Mexico and southwestern USA in spring of 2009 [1], [2]. The disease was next observed in other parts of the USA in late March 2009 and the virus was first isolated in mid April 2009 [3], [4]. Throughout the spring, the epidemic spread worldwide and the World Health Organization (WHO) declared an influenza pandemic, phase 6, on 11 June 2009 [5]. By 25 April 2010, 17, 919 deaths attributable to H1N1 S-OIV were reported with confirmed cases in more than 214 countries [6].

Influenza is a negative sense single-stranded RNA virus in the family *Orthomyxoviridae*. It is maintained in migratory waterfowl and can infect many species including humans, birds, pigs, horses, and other animals. The virus genome contains eight segments that code for 10 or 11 proteins. Three segments encode the polymerase complex: basic polymerase 2 (PB2), basic polymerase 1 (PB1), and the acidic protein (PA). The nucleoprotein segment (NP) encodes a protein that binds to viral RNA. The matrix segment (MP) encodes two proteins: a structural component of the viral capsid and a membrane ion channel. The nonstructural segment (NS) encodes a protein essential for cellular RNA processing and transport. Two other segments, hemagglutinin (HA) and neuraminidase (NA), encode viral surface glycoproteins responsible for host cell entry and exit, respectively [7], [8]. The hemagglutinin and neuraminidase genes determine the viral subtype, designated ${\rm H}x{\rm N}y$, where $x$ is one of 16 known hemagglutinin subtypes [9] and $y$ is one of nine known neuraminidase subtypes [7].

When two different influenza viruses infect the same host cell, novel combinations of the eight genomic segments can create a novel virus through a process known as reassortment [7], [8], a form of horizontal gene transfer (HGT). Often, the host population is immunologically naïve to the new viruses created through reassortment, which can lead to worldwide pandemics as observed in 1957 with the H2N2 pandemic, in 1968 with the H3N2 pandemic, and in 2009 with the H1N1 pandemic [8], [10]. The role of reassortment in influenza evolution is also discussed in [11] and [12].

Reassortment events leading to novel influenza viruses are poorly understood, greatly underestimated, and thus are an area of continuing research. Reassortment events are often identified by visual comparisons of incongruence among phylogenetic trees [13]. Some algorithms have been developed to infer reassortment events through statistical techniques in the absence of phylogenetics [14], [15]. Prior research in using phylogenetic networks to analyze situations where HGT occurs has been done by Huson and Bryant [16], and Makarenkov and Legendre [17]. An extensive survey of combinatorial methods for phylogenetic networks appears in [18].

In this paper, we build on previous work by Bokhari and Janies [19] to explore the evolution of H1N1 S-OIV pandemic viruses using a reassortment network. This paper reports multidisciplinary research, involving several diverse fields. As such, we must necessarily cover a wide range of issues in order to provide a clear understanding of our methods and results. It is important to describe many of the low-level implementation details to allow other researchers to appreciate the complexities of this project and pursue its applications and extensions. In particular, we cover the details of data gathering and processing that were necessary to create the database that is at the center of this work. The issues of parallel computation, both on a distributed memory commodity cluster as well as on a shared-memory massively multithreaded supercomputer are also covered, as the reassortment algorithm could not be run in an acceptable amount of time without these. Finally, the presentation of output as “in-trees” that capture both mutation and reassortment is important for interpretation and evaluation of our results.

The main contributions of this paper are:

1. Implementation of a parallel version of the Bokhari and Janies [19] reassortment algorithm.
2. Testing of this algorithm on all 5, 016 fully sequenced influenza A genomes, as of late 2009.
3. Validation of the reassortments reconstructed by the network algorithm against prior results by other researchers.
4. Identification of six “bottleneck viruses” that lie on almost every evolutionary path to the 2009 S-OIV set of viruses. These possibly played a significant role in the S-OIV pandemic and are worthy of further study in a molecular biology context.

We start the paper with an overview of the workflow of our research in Section 2, which is followed by a discussion of the architecture of the Cray XMT supercomputer. In Section 3, we review existing models for S-OIV reassortment. The main results of our paper appear in Sections 4 and 5. We conclude with a discussion of our results and suggestions for future research in Section 6. ^1^^1.^The Supplemental Material to this paper (which can be found on the Computer Society Digital Library at http://doi.ieeecomputersociety.org/10.1109/TCBB.2011.95) includes a set of detailed Appendices. It also contains several large pdf files that are electronically annotated. The process of uploading may not have preserved the annotations. All Supplemental Material is duplicated at bmi.osu.edu/~shahid/SOIV, where the files have been verified to behave correctly. Annotations can only be viewed with the Adobe Reader.

## 2 Overview of Workflow

**Fig. 1.**
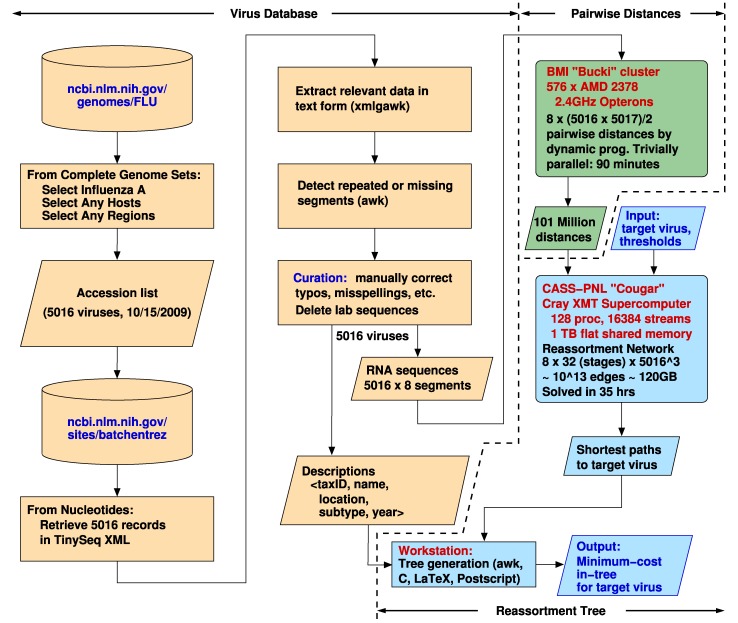
Workflow: XML = Extensible Markup Language, tinySeq XML = a lightweight version of XML designed for DNA sequences, awk = a pattern scanning and matching language, xmlgawk = an enhanced version of awk that can handle XML, and BMI = Biomedical Informatics, The Ohio State University (bmi.osu.edu), CASS-PNL = Center for Adaptive Supercomputing Software, Pacific Northwest National Laboratory (cass-mt.pnl.gov).

There are three main components of our research (Fig. 1). To start with, virus names, dates, locations, subtypes, and nucleotide sequences were downloaded from the National Center for Biotechnology Information (www.ncbi.nlm.nih.gov). Next, pairwise distances between all pairs of corresponding sequences were computed using dynamic programming. Finally, the distances were used to create a Reassortment Network and to compute the minimum cost paths from all viruses to a given target virus. (A minimum cost path, or shortest path, from node $s$ to node $t$ has the shortest distance over all paths from $s$ to $t$.) These shortest paths are used to create the in-tree that has, in general, all known viruses as its leaves and the given target virus as its root.

### 2.1 Creating the Virus Database

Although the RNA sequences of influenza viruses are available in the public databases www.ncbi.nlm.nih.gov/genomes/FLU [20] and www.ncbi.nlm.nih.gov/sites/batchentrez, extracting this information in a form that could be used for our study was a challenging problem that required significant manual curation.

We need, for each virus, a unique strain-specific identifier (a TaxID), strain name (e.g., A/Canada-NS/RV1535/2009(H1N1)), subtype (H1N1 in this case), year (2009), and RNA sequences for each of the eight segments. Acquiring this information is complicated by the following issues:

• Many segment sequences are repeated in the database due to redundant effort.
• The year entry in the database is often not standardized (e.g., 1979-chicken instead of 1979).
• Sequences include those isolated from laboratory adapted and vaccine strains that have to be excluded by inspection.
• Segment types are part of name strings that also include place names. For example, “PA” could be a location (a state of the US) or the segment type.
• Segment types are labeled in numerous ways, e.g., polymerase 2, polymerase basic protein 2, segment 1, Sequence 1, P2, PB2, pb2.

These issues make it difficult to fully automate the process of extracting information from the database and require significant manual effort. Full details of the steps required to acquire this data are given in the Supplemental Material, Appendix E, which can be found on the Computer Society Digital Library at http://doi.ieeecomputersociety.org/10.1109/TCBB.2011.95. Our final curated list of viruses is available in the supplemental material as http://bmi.osu.edu/~shahid/SOIV/CuratedList.txt.

### 2.2 Notation (Adapted from [19])

$n$ The number of viruses.
$\tau$ The number of stages of evolution.
$s$ The number of segments in each virus.
$v[\sigma ]$ Segment number $\sigma$ of virus $v$.
$\omega (i, j)[\sigma ]$ The *distance* between segments $\sigma$ of
viruses $i$ and $j$.
${\cal W} (i, j)$ The *distance* between viruses $i$ and $j$.
${\cal W}$ $(i, j) = \sum_{\psi =1}^{s}\omega (i, j)[\psi ]$.
$i \;\leftarrow \; j[\sigma ]$ A reassorted virus where segment $\sigma$ of
virus $j$ replaces segment $\sigma$ of virus $i$.
${\cal W}$ $(i\;\leftarrow \; j[\sigma ], k)$ The *distance* between a reassorted virus
$i\;\leftarrow \; j[\sigma ]$ and virus $k$.
${\cal W} \;(i\;\leftarrow \; j[\sigma ], k) = \sum_{\psi =1}^{s}\omega (i\;\leftarrow \; j[\sigma ], k)[\psi ]$.

### 2.3 Computing Intersegment Distances

Weighted edit distances for every pair of corresponding segments were computed using standard dynamic programming [21, Section 11.5], as detailed in [19]. We use “end-space free” alignments [21 Section 11.6.4]. Such alignments ignore any missing bases at the ends of the input strings, which are due to sequencing artifact. When computing distances, we needed to account for symbols other than A, C, T, G, that represent ambiguous bases [22]. This issue is described in detail in the online Supplemental Material, Appendix F.

The number of fully sequenced (all eight segments known) influenza A viruses in www.ncbi.nlm.nih.gov/genomes/FLU as of 15 October 2009 was 5, 016. Thus, we needed to carry out $8\times (5{,}016\times 5{,}017)/2\approx 101\times 10^6$ alignments. The memory requirements in this case are modest, as the length of an influenza segment is ${<} 2{,}500$ bases, implying dynamic programming matrices of size ${<}10^7$. Furthermore, the $101\times 10^6$ alignments are completely independent and thus trivial to parallelize.

We initially used the resources of Ohio Supercomputer Center (OSC, www.osc.edu), which has several thousand processors. The computation of all distances took 12-24 hours on this shared system, where we had to compete with hundreds of other users.

During the course of our research, our department (Biomedical Informatics) acquired its own cluster, called “Bucki,” with 576 AMD Opteron 2378 (2.4 GHz) processors that we could use in dedicated mode. The time on this system was reduced to 90 minutes by finely dividing the workload, as detailed in the online Supplemental Material, Appendix G.

### 2.4 The Reassortment Network

We now turn to the actual creation and solution of reassortment networks. As the theory underlying these networks has been discussed in detail by Bokhari and Janies [19], we present only a brief sketch.

A reassortment network representing $\tau$ stages of evolution has $2\tau +1$ layers $0\ldots 2\tau$. Even layers represent viruses and odd layers represent events, which can be *stasis*, *mutation*, or *reassortment*. Edges extend only from layer $i$ to $i+1$, $0\le i <2\tau$ (resulting in a *multipartite* graph). A path from a virus $v$ in the first layer to a virus $u$ in the last layer represents a series of stasis, mutation, or reassortment events that transform $v$ to $u$.

Edges in the reassortment network have weights on them. The weighting scheme is designed such that the cost of the path between virus $v$ in layer 0 and virus $u$ in layer $2\tau$ equals the sum of the costs of the mutation and reassortment events that transform $v$ into $u$ (stasis costs between identical segments are, of course, zero).

The cost of a mutation of virus $i$ into virus $k$ is the sum of the edit costs of the individual segments. When virus $i$ reassorts to obtain a segment $\sigma$ from virus $j$ to become virus $k$, the reassortment cost is the sum of the segment edit distances between virus $i$ (with segment $\sigma$ replaced by the corresponding segment of virus $j$) and virus $k$. These concepts are clarified in Fi gs. 12 and 13 in the online Supplemental Material, Appendix D.

### 2.5 Motivation for Reassortment Algorithm

Our reassortment network is a layered graph in which alternating layers of nodes correspond to viruses and reassortment events between pairs of viruses. Edges in this graph correspond to transitions between viruses and reassortment events, with edge weights corresponding to the costs of transitions. These costs are the sums of segment distances between pairs of viruses.

Paths in a reassortment network correspond to evolutionary changes and the lengths of the paths correspond to the sums of the edge weights in the paths. Lower path lengths correspond to smaller sums of distances between the corresponding sequence of viruses. It follows that the shortest path indicates the minimum cost sequence of mutation and/or reassortment events required to transform one virus into another.

### 2.6 Finding Shortest Paths

Once a $\tau$ stage reassortment network for $n$ viruses with $s$ segments each has been set up, the shortest evolutionary path between any two viruses can be found in time ${\cal {O}}(\tau s^2n^3)$. This time suffices to find shortest paths from all viruses in layer 0 to a given target virus $t$ in layer $2\tau$. These paths constitute the *in-tree* for the target virus. The expression ${\cal {O}}(\tau s^2n^3)$ represents a very large number of computational steps, given that $\tau \approx 30, s=8$, and $n\approx 5{,}000$, and massive parallelism is required to achieve reasonable run times, as discussed below.

### 2.7 The Cray XMT

The algorithm for finding shortest paths in the reassortment network is difficult to parallelize on conventional cluster machines because

1. the reassortment network is a graph that has little locality and thus requires long-range communications between graph nodes,
2. there is a strict precedence relationship on the order in which the nodes are labeled, that is, from layer 0 to layer $2n$,
3. if layers are assigned to individual processors, then all processors whose layers are currently not being updated will be idle, and
4. if layers are partitioned over processors, then there will be need for frequent and heavy interprocessor communications.

The Cray Extreme Multithreading (XMT) supercomputer is the latest in a family of machines that originated in the Tera [23] and subsequently evolved into the MTA (Multithreaded Architecture) [24]. Reviews of the architecture of this machine and its applications are available in [25] and [26].

The key features of this machine include

1. hardware support for 128 threads per processor,
2. zero overhead switching between threads,
3. large uniformly accessible shared memory,
4. extremely fine grained synchronization using Full/Empty bits with individual 64-bit words,
5. powerful interconnect, and
6. powerful compiler and performance analysis tools.

These features of the XMT allow straightforward and efficient parallelization of many important problems, especially those in bioinformatics, as surveyed in [26]. In particular, the XMT is the only machine that can efficiently parallelize large-scale reassortment networks.

The largest XMT that is available to researchers as of 2010 is the 128 processor, 1 Terabyte machine at the Center for Adaptive Supercomputer Software (CASS) in the Pacific Northwest National Laboratory (PNL).

### 2.8 Parallelization of Algorithm

The XMT allows the parallelization of ordinary C code with some loop restructuring and the judicious addition of pragmas (compiler directives in code). The use of machine specific synchronization is occasionally required.

**Fig. 2.**
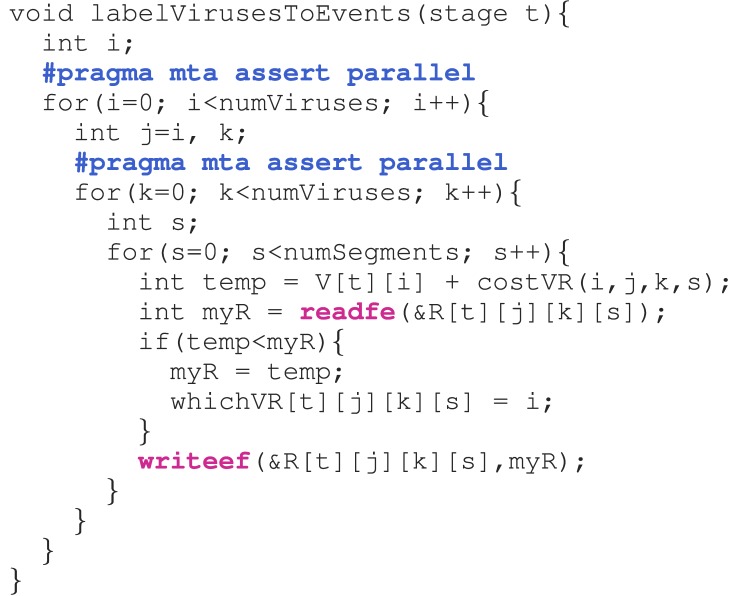
The code for labeling events from viruses.

As an example, Fig. 2 describes the routine that labels event nodes from virus nodes when finding shortest paths in a reassortment network. The **#pragmas** assure the compiler that the loops that follow are safely parallelizable. Only the i and k loops need to be parallelized since the number of ${\rm viruses} = 5{,}016$ and this results in enough parallelism ($5{,}016^2$ computations) to keep the machine busy. Thus, the s loop is not parallelized. The **readfe(.)** (wait until full, then read and set empty) (**writeef(.)** (wait until empty, then write and set full)) functions lock (unlock) their arguments (which are *individual words*) so that multiple threads can safely and correctly update them. Although expressed as functions, these are really machine operations that are executed in one clock cycle, leading to very efficient, fine grained synchronization. This behavior is possible because the Cray XMT supercomputer has special synchronization bits with *each* memory location. An illustration of synchronization is provided in the online Supplemental Material, Appendix H. Notice how variables are declared only in the scopes where they are used. This ensures that if a loop is parallelized, the corresponding thread will have an independent copy of the variable, thus eliminating contention.

### 2.9 Implementation Details

When going from a serial to a parallel version of our code, we first examined the major loops to identify which could be parallelized. In these loops, we had to ensure that all accesses to shared variables were safely parallelizable. This is easy to do, since the XMT compiler will indicate unsafe parallelization (i.e., situations where there are accesses to shared locations without appropriate synchronization). Full/Empty operations, **readfe(.)** and **writeef(.)**, as described above, were used in such situations. In a few cases, loops had to be restructured so as to allow efficient parallelization.

One of the major issues when using the XMT is the speed of input/output. When large volumes of data, such as the distance matrices (which are of size 101 million words) are to be input, these have first to be loaded into a special parallel file system and then transferred in parallel into the 1 Terabyte shared memory of the XMT. Ordinary C input/output functions are executed serially and cannot be used for large data volumes.

Our code occupied about 120 GB of memory and ran at the rate of about 1.1 hours per stage, for a total of 35 hours for a 32-stage problem, using 128 processors.

## 3 S-OIV Evolution Models

**Fig. 3.**
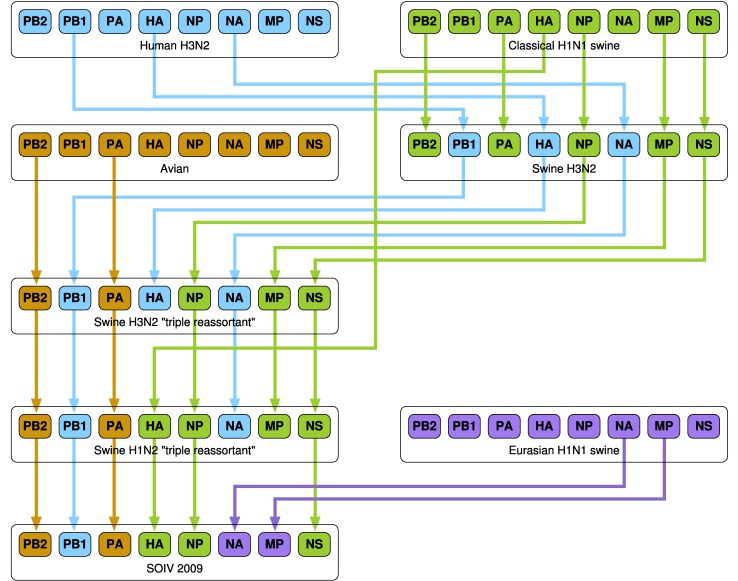
A model of influenza evolution based on [27] and [29].

**Fig. 4.**
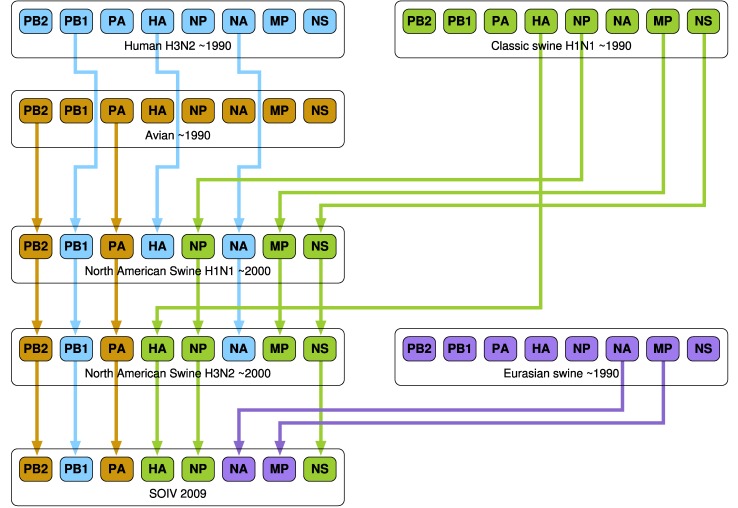
The model presented by Trifonov et al. [28] is a subset of the model in Fig. 3; it omits a stage of swine H3N2 viruses.

**Fig. 5.**
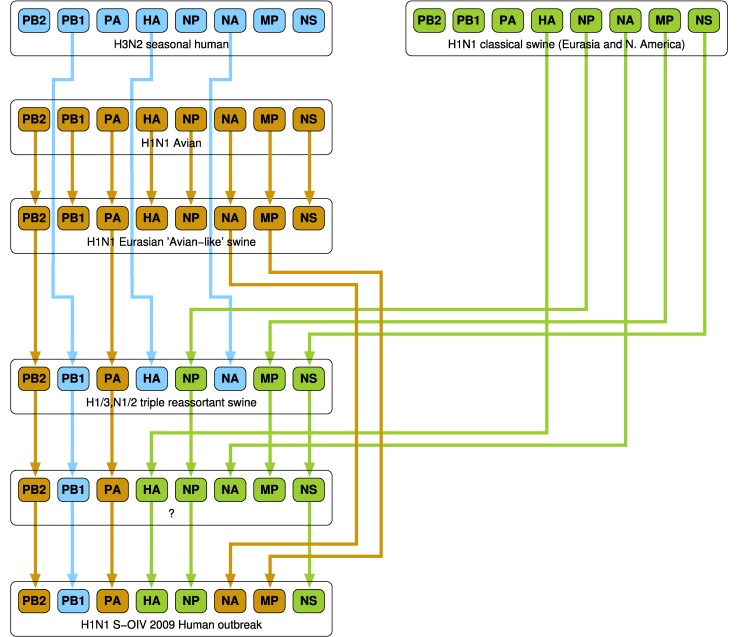
The model of Smith at al. [30] differs significantly from the preceding two models. Here, “Eurasian H1N1 swine” do not stand in isolation but are derived from Avian H1N1.

A number of research groups have presented models of the emergence of influenza in human hosts from influenza in swine hosts, including S-OIV. These include [4], [27], [28], [29], [30]. Figs. 3, 4, and 5 present the salient features of these models.

In work by Dawood et al. [4], Olsen [27], Trifonov et al. [28], and Kingsford et al. [29], “classes” of viruses such as “Classical H1N1 swine,” “Eurasian H1N1 swine,” etc. are mentioned. We have been unable to find enumerated lists of these classes of viruses in the literature. This lack of specificity complicates the comparison of the results of our algorithm with those of other research groups.

Consider class “Eurasian Swine Influenza” shown in of [29], Fig. 3], which is a phylogenetic tree for the NA segment and includes many avian viruses, e.g., A/duck/Nanchang/1904/1992, A/goose/Italy/296426/2003, A/chicken/ Hebei/718/2001, etc. The rationale behind this inclusion is not explained and appears to be based only on the similarity between the NA segments and not on any clinical evidence of infection of swine with avian viruses.

## 4 Validation of Model

We ran our reassortment network for different target viruses. To start with, we present the results of two runs with non-SOIV viruses as targets. The objective here is to test our algorithm against prior results by Karasin et al. [31]. The following validation covers virus evolution over the period 1977-1999, while the S-OIV investigation (Section 5, below) covers the years 1930-2009. The same master database of intersegment distances was used in both cases; the reassortment algorithm is insensitive to the year of the target virus.

### 4.1 Constraints

We used a threshold of 500: if two viruses differed in more than distance 500 in any one segment, the mutation or reassortment event was ignored. This is necessary to get useful information from our reassortment network. This is because the algorithm searches for shortest paths in terms of sums of edge weights. If a high threshold (or no threshold) is used, all evolutionary paths will be only a few edges long, thus obscuring fine grained information on mutations and reassortments. If a zero threshold is used, no path will be found (as the target will be disconnected from the rest of the graph). For our 5, 016 virus, 35 stage problem, we found that 500 was a choice of threshold that gave useful information. This phenomenon is discussed in Section 5.4 and Fig. 8 of our previously published paper [19].

We allowed reassortments between viruses $i$ and $j$ to yield $k$ only if the years of $i$ and $j$ were ≤ the year of $k$. Under these constraints, some viruses may not participate in paths to the target.

### 4.2 Interpreting In-Trees

**Fig. 6.**
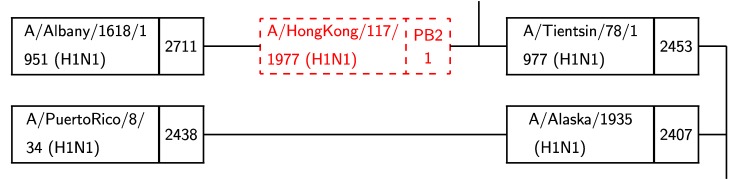
A reassortment (top) and a mutation (bottom).

As described in Fig. 6, a black box represents a virus with the number on the right of the box indicating the length of the shortest path from that virus to the target. Two black boxes connected by a black line indicate a mutation event. Each dashed red box indicates a reassortment, with the segment name and number indicated on the right hand side. In Fig. 6, A/PuertoRico/8/34 (which has total distance 2, 348 from the target (not shown)) mutates into A/Alaska/1935 which is at distance 2, 407 from the target. A/Albany/1618/1951 reassorts: it obtains segment 1 (PB2) from A/HongKong/117/1977 to become A/Tientsin/78/1977.

Virus names that are too long to fit in available space are truncated (indicated with “${\ast}$ ”) and full names are available in the corresponding digital annotations in the pdf files in the online Supplemental Material.

### 4.3 A/Swine/Colorado/1/1977

**Fig. 7.**
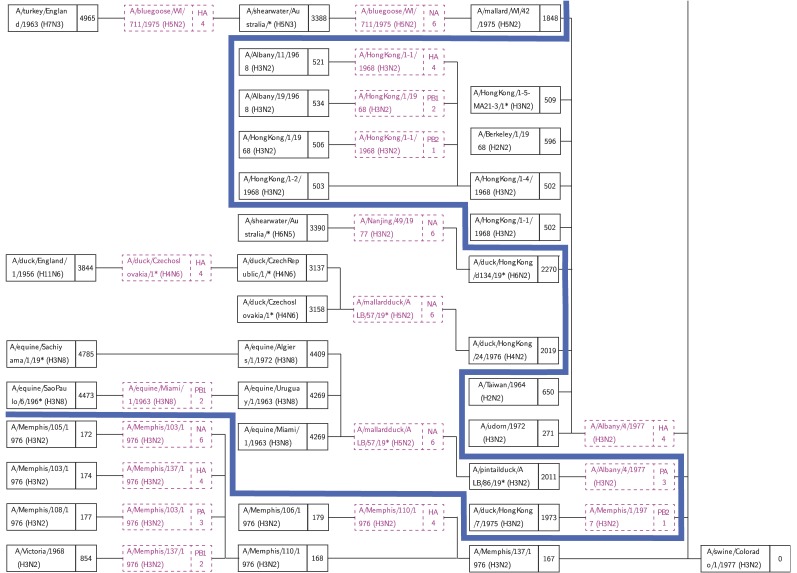
Part of the in-tree for target A/swine/Colorado/1/1977(H3N2). There is a clear differentiation between human and nonhuman ancestors (blue cut).

Fig. 7 shows part of the tree resulting from a run with A/swine/Colorado/1/1977(H3N2) as target. The full tree is in Supplemental file ASwineColorado.pdf. The blue cut associates (on its right hand side) viruses that are distance ${\le} 900$ from the target virus. These viruses are exclusively of human origin. Nonhuman origin viruses, on the left side of the cut, have weight greater than 1, 800. There is a clear differentiation between human (${<} 900$) and nonhuman (${>}1{,}800$) viruses. This validates the result by Karasin et al. [31] that A/swine/Colorado/1/1977 is wholly human in origin.

### 4.4 A/Swine/Nebraska/209/98

**Fig. 8.**
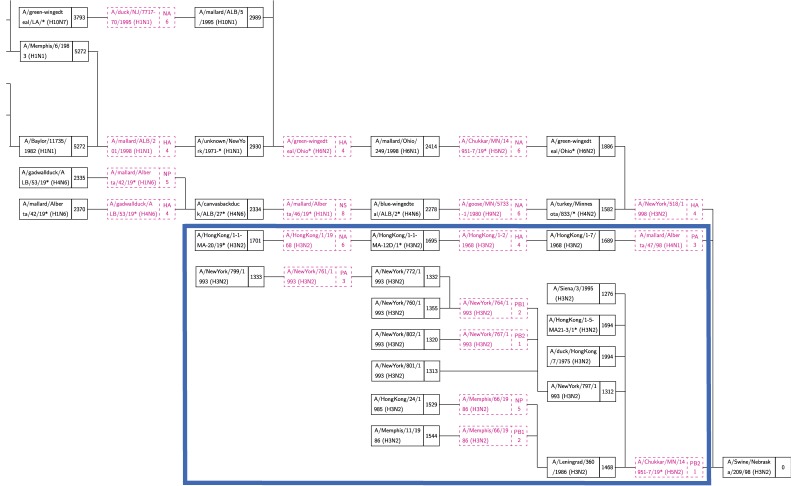
Part of the in-tree for A/swine/Nebraska/209/98(H3N2). The lowest cost paths (blue box) include reassortments with avian viruses to obtain PA and PB1.

The in-tree from a run with target A/swine/Nebraska/209/98(H3N2) is shown in Fig. 8. The full tree is in Supplemental file ASwineNebraska.pdf. According to Karasin et al. [31], the PA and PB2 segments should be derived from avian viruses and the remaining from human. In the tree of Fig. 8, the lowest cost paths (blue box) pass through reassortments with avian viruses to obtain PA and PB2 just before the target. This partially supports Karasin et al. [31], since these are two disjoint sets of paths and not one path with two reassortments, as we would have expected. We conjecture that this is due to missing data—a richer data set might have yielded the expected path.

## 5 Results

### 5.1 Parameters of 32-Stage Run

We now present the results of a run with the exemplar S-OIV virus A/California/04/2009 as target. This 32-stage run took 35 hours on a 128 processor Cray XMT. The reassortment network generates an in-tree of shortest paths from 5, 015 viruses to A/California/04/2009.

In addition to the constraints stated in Section 4.1, we used a reassortment overhead of 10 units to suppress trivial, small distance reassortments that would otherwise clutter up our paths. Such reassortments are indistinguishable from point mutations and drive interesting large-distance reassortments out of the 35-stage range of our experiment. The overhead is incorporated by adding 10 units to the reassortment edges of our network.

### 5.2 Suppressing Intra-S-OIV Events

As a result of the intense interest in the pandemic strains, the number of S-OIV viruses in our database is a disproportionately large fraction of the total viruses. Consequently, the target virus is surrounded by a very large subtree that represents *intra*-S-OIV evolution and obscures other evolutionary events. To concentrate on our immediate objective of tracing the origin of the exemplar S-OIV virus A/California/04/2009, we suppressed paths through all other S-OIVs by temporarily setting their distances from everyone else to infinity.

### 5.3 Viewing the In-Trees

Despite the constraints mentioned in Section 4.1, the size of the database is such that very large trees are still generated and it is a challenge to visualize the results. After trying several approaches, we have chosen to separate the in-trees by the years of their source viruses. Thus, F ig. 14 in the online Supplemental Material, Appendix J, shows the in-tree for viruses from the 1930s, 40s, 50s while F ig. 15 shows those from the 1960s (trees for subsequent decades are available in the online Supplemental Material). For clarity, even with this approach, we needed to prune paths in which the leaf nodes were highly similar in terms of their distance to target. As the number of sequenced viruses has been increasing dramatically since the 1960s, it is impossible to show results from the 1970s onward on paper. Full information from the run is available as a spreadsheet as described below.

In Supplemental Fi g. 14, we show part of the in-tree corresponding to source viruses from the 1930-59 time period. The paths in this figure indicate how these viruses could possibly have evolved into the S-OIV A/California/04/209.

Although the source viruses (leaf nodes) are 60 or more years old, several have low-distance paths to the S-OIV.

In particular, A/swine/1931, could have reassorted with A/swine/Kansas/015252 to eventually become A/California/04/2009. A/chicken/Germany/N/1949 has high distance to the target, but has a path that transforms it into A/California/04/2009 after three reassortments. The intermediate viruses in this path are evenly spaced in terms of distance.

Note that many of the paths pass through A/swine/HongKong/1562/2005(H1N2), A/swine/Guangxi/13/2006(H1N2), and A/swine/Kansas/77778/2007(H1N1). These viruses are two edges away from the target, have path weight ${\le} 1{,}010$ and form part of an important “bottleneck” set that we will discuss below. The annotated tree is in Supplemental file tree304050.pdf.

The in-tree for the 1960s is shown in Supplemental Fi g. 15. It is noteworthy that the bottleneck set has now expanded to include A/swine/HongKong/1110/2006(H1N2) and that most paths pass through this set. The annotated tree is in Supplemental file tree60.pdf. Supplemental file tree70.pdf holds the in-tree for the 1970s. The bottleneck viruses are the same as for the 1960s, and have path weight to target ${\le} 1{,}010$. A number of additional viruses now occur at distance two edges from the target, but these have path weight ${>}2{,}000$, which is significantly greater than the bottleneck viruses. For the 1980s, Supplemental file tree80.pdf has a new bottleneck virus: A/swine/ Shanghai/1/2007. This has slightly greater distance, i.e., 1, 030, than the others, but is still significantly smaller than other viruses at distance two edges from target. Supplemental file tree90.pdf shows a new bottleneck virus A/Iowa/CEID23/2005(H1N1), with distance 1, 008 to target.

**Fig. 9.**
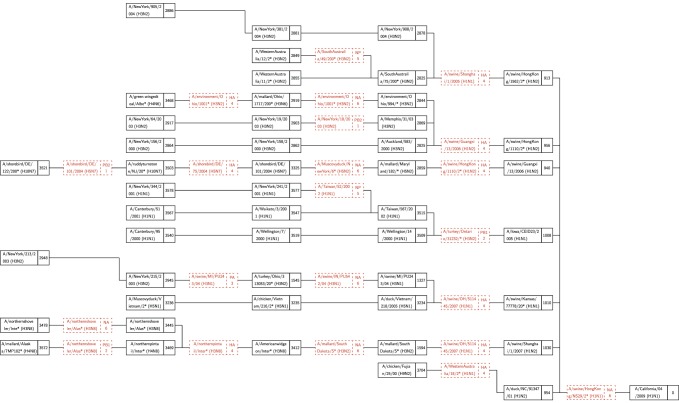
A subset of the in-tree for A/California/04/2009 that shows only source viruses (i.e., leaf nodes) from 2000 to 2009. The first six of seven viruses in the rightmost column are “bottleneck” viruses, with hundreds of paths through them. Only a few representative paths are shown. Black boxes represent viruses; red boxes stand for reassortment events (see Fig. 6). A/duck/NC/91347/01 is *not* a bottleneck, as discussed in text.

For viruses that were identified in 2000 and later, an annotated tree is available in Supplemental file tree00.pdf. Fig. 9 shows a selection of paths that pass through a set of six bottleneck viruses, five of which were encountered in previous decades. The new bottleneck is A/swine/Shanghai/1/2007(H1N2).

The virus A/duck/NC/91347/01(H1N2) is located in the same position in the tree as the bottleneck viruses and has distance to target of 954, which is smaller than any of the bottlenecks. However, in our run, only one path is found through it, unlike the bottleneck viruses which have hundreds of paths, as described below. We, therefore, do not include this in our list of bottleneck viruses, but do consider it worthy of future analysis in a molecular biology context because of its low distance.

### 5.4 Bottleneck Viruses

Analysis of the results of the 32-stage run reveals that 3, 600 out of 3, 926 paths pass through a set of bottleneck viruses before reaching the S-OIV A/California/04/2009, as shown in Table 1. These viruses are:

1. A/swine/Shanghai/1/2007(H1N2)
2. A/swine/Guangxi/13/2006(H1N2)
3. A/swine/HongKong/1110/2006(H1N2)
4. A/swine/HongKong/1562/2005(H1N2)
5. A/swine/Kansas/77778/2007(H1N1)
6. A/Iowa/CEID23/2005(H1N1)
  All of the above reassort with
7. A/swine/HongKong/NS29/2009(H1N1)

to obtain the NA segment before reaching A/California/04/2009. Viruses 1-6 are obtained by reassortments with a number of different viruses. Some of these are the bottlenecks themselves. For example, one of the possible evolutionary paths in Fig. 9 shows A/mallard/Maryland/182/2006 reassorting with A/swine/HongKong/1110/2006 (bottleneck no. 3) to yield A/swine/Guangxi/13/2006 (bottleneck no. 2). The *nonbottleneck* viruses that donate segments are:

1. A/swine/OH/511445/2007(H1N1)
2. A/turkey/Ontario/31232/2005(H3N2)
3. A/swine/Shanghai/1/2005(H1N1)

**Fig. 10.**
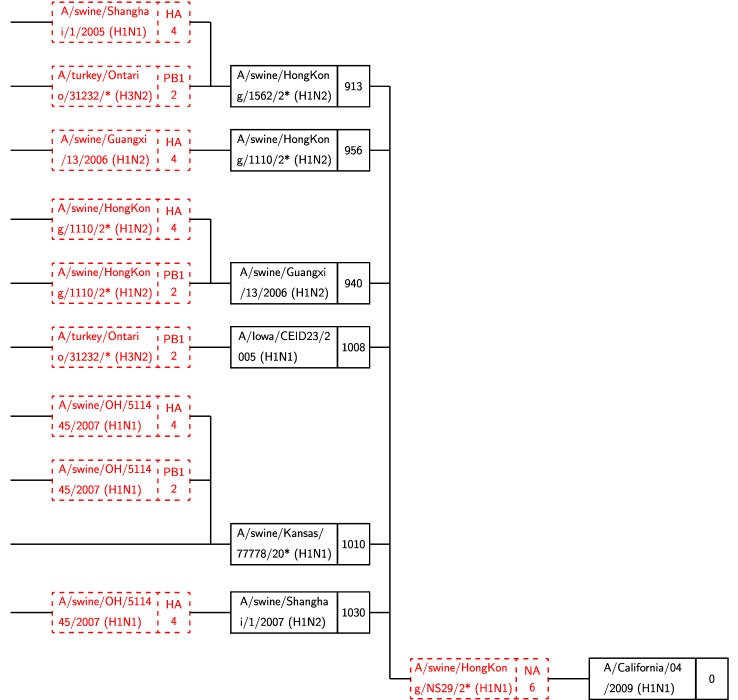
The six bottleneck viruses (Column 2) reassort with A/swine/HongKong/NS29/2009 to reach the target.

Fig. 10 illustrates the bottleneck viruses. This subtree was obtained from the full tree by only including paths that pass through bottleneck viruses 1-6, listed above, and excluding everything beyond the second reassortment.

**TABLE 1:** Numbers of Paths through Bottleneck Viruses

1	A/swine/Shanghai/1/2007	292
2	A/swine/Guangxi/13/2006	1252
3	A/swine/HongKong/1110/2006	199
4	A/swine/HongKong/1562/2005	919
5	A/swine/Kansas/77778/2007	736
6	A/Iowa/CEID23/2005	202
Paths through bottleneck viruses		3600
Total paths in tree		3926

A very large proportion of paths reach the S-OIV target through the bottleneck viruses, as shown in Table 1. This strongly suggests that these paths represent actual evolutionary events. Note, however, that only a subset of these paths is shown in the figures discussed above. The fact that these six bottleneck viruses occur repeatedly and at the lowest distance found from the target A/California/04/2009 suggests that they are important in the evolutionary history of S-OIV influenza A.

### 5.5 Spreadsheet

The Supplemental Material includes a spreadsheet SOIVspread.xls that lists *all* paths to A/California/04/2009, from the 32-stage run, sorted by year of source virus and then by path cost. Bottleneck viruses are marked with “${\ast}{\ast}{\ast}$ ” in this spreadsheet and in the tables that follow below. Explanation of the notation used in this spreadsheet appears in the Supplemental Appendix I.

## 6 Discussion

Our analysis yields detailed information on the series of reassortments and mutations required to transform a set of viruses to S-OIV viruses. We have identified six bottleneck viruses that almost invariably occur on shortest paths found to the target S-OIV virus.

To investigate these bottleneck viruses further, Table 2 shows details of the reassortments that occur in the final stage of the network. The entries in this table can be interpreted using the notation given in Section 2.2. As an example, the first block shows virus 1, A/swine/ Shanghai/1/2007(H1N2) reassorting with virus 7 A/swine/HongKong/NS29/2009(H1N1) to yield target $t$ A/California/04/2009(H1N1). The row $w(1, 7)$ 472 422 396 574 335 1029 143 171 (3542) indicates the distances between the eight segments of viruses 1 and 7, with the number in parenthesis (3542) giving the sum of these distances. Similarly, $w(7, t)$ and $(1, t)$ indicate the distances between viruses 7 and 1, and target $t$, respectively. $w(1\;\leftarrow \; 7[6], t)$ shows the distances between the reassorted virus (i.e., the virus obtained by replacing segment 6 (NA) of virus 1 with the corresponding segment of virus 7) and the target virus $t$. Further elucidation of the reassortment notation is available in the Supplemental Appendix C.

**TABLE 2:** Reassortments of Bottlenecks 1-6 with Virus 7 to Obtain Target $t$

	PB2	PB1	PA	HA	NP	NA	MP	NS	$\sum$
1	A/swine/Shanghai/1 /2007(H1N2)								
$w (1, 7)$	472	422	396	574	335	1029	143	171	(3542)
7	A/swine/HongKong/NS29/2009(H1N1)								
$w(7, t)$	472	410	397	601	331	160	53	192	(2616)
$t$	A/California/04/2009(H1N1)								
$w(1, t)$	126	143	123	202	66	1041	144	56	(1901)
$w\left ( 1\leftarrow 7\left [ 6 \right ], t \right )$	126	143	123	202	66	160	144	56	(1020)
2	A/swine/Guangxi/13/2006(H1N2)								
$w (2, 7)$	475	449	455	632	379	1047	148	177	(3762)
7	A/swine/HongKong/NS29/2009(H1N1)								
$w(7, t)$	472	410	397	601	331	160	53	192	(2616)
$t$	A/California/04/2009(H1N1)								
$w (2, t)$	114	124	143	111	70	1064	149	59	(1834)
$w\left ( 2\leftarrow 7\left [ 6 \right ], t \right )$	114	124	143	111	70	160	149	59	(930)
3	A/swine/HongKong/1110/2006(H1N2)								
$w (3, 7)$	458	416	397	593	337	1026	148	174	(3549)
7	A/swine/HongKong/NS29/2009(H1N1)								
$w(7, t)$	472	410	397	601	331	160	53	192	(2616)
$t$	A/California/04/2009(H1N1)								
$w(3, t)$	125	128	131	116	81	1031	143	62	(1817)
$w\left ( 3\leftarrow 7\left [ 6 \right ], t \right )$	125	128	131	116	81	160	143	62	(946)
4	A/swine/HongKong/1562/2005(H1N2)								
$w (4, 7)$	463	411	394	584	338	1059	147	175	(3571)
7	A/swine/HongKong/NS29/2009(H1N1)								
$w(7, t)$	472	410	397	601	331	160	53	192	(2616)
$t$	A/California/04/2009(H1N1)								
$w(4, t)$	108	123	124	102	73	1076	148	65	(1819)
$w\left ( 4\leftarrow 7\left [ 6 \right ], t \right )$	108	123	124	102	73	160	148	65	(903)
5	A/swine/Kansas/77778/2007(H1N1)								
$w (5, 7)$	458	395	389	597	338	361	146	192	(2876)
7	A/swine/HongKong/NS29/2009(H1N1)								
$w(7, t)$	472	410	397	601	331	160	53	192	(2616)
$t$	A/California/04/2009(H1N1)								
$w (5, t)$	135	132	139	127	95	362	148	64	(1202)
$w\left ( 5\leftarrow 7\left [ 6 \right ], t \right )$	135	132	139	127	95	160	148	64	(1000)
6	A/Iowa/CEID23/2005(H1N1)								
$w (6, 7)$	465	414	395	592	324	372	151	175	(2888)
7	A/swine/HongKong/NS29/2009(H1N1)								
$w(7, t)$	472	410	397	601	331	160	53	192	(2616)
$t$	A/California/04/2009(H1N1)								
$w(6, t)$	124	129	118	184	76	363	153	54	(1201)
$w\left ( 6\leftarrow 7\left [ 6 \right ], t \right )$	124	129	118	184	76	160	153	54	(998)

In Table 2, all bottleneck viruses have small differences from the target virus in all segments except NA. A reassortment with A/swine/HongKong/NS29/2009, which has low distance (160) for the NA segment results in a virus that is very close to the target. Furthermore, the years of the three viruses in each possible reassortment are obviously consistent (as a result of constraints built into the reassortment network). Finally, most of these viruses are remarkably close to each other, as Table 3 demonstrates. This distance matrix gives the absolute distances (sums of the segment-wise distances) between all pairs of bottleneck viruses. The four H1N2 viruses are very close to each other as are the two H1N1 viruses.

**TABLE 3:** Absolute Pairwise Distances between Bottleneck Viruses

1	2	3	4	5	6	
0	554	541	487	1706	1662	1
	0	473	426	1735	1753	2
		0	349	1643	1682	3
			0	1625	1666	4
				0	654	5
						0

Tables 4 and 5 show the shortest paths from our run, organized by number of edges and permit us to identify unusual events. We would expect the path length to increase with the number of edges in a path across paths and within paths and this does, in general, happen. However it is noticeable that the weight of the shortest path for edges $=2$ in Table 4 is remarkably small. This path starts in A/duck/NC/91347/01, a virus that we have already noted, in Section 5.3, as being oddly similar to the bottleneck viruses (though only having a single path through it). Olsen et al. [32] described this strain in 2003 and showed that it has great similarity with swine origin viruses. Similarly, for paths with three edges, A/swine/OH/511445/2007 (*not* a bottleneck) has an unusually low distance to the target, compared with other viruses with the same number of edges.

**TABLE 4:**
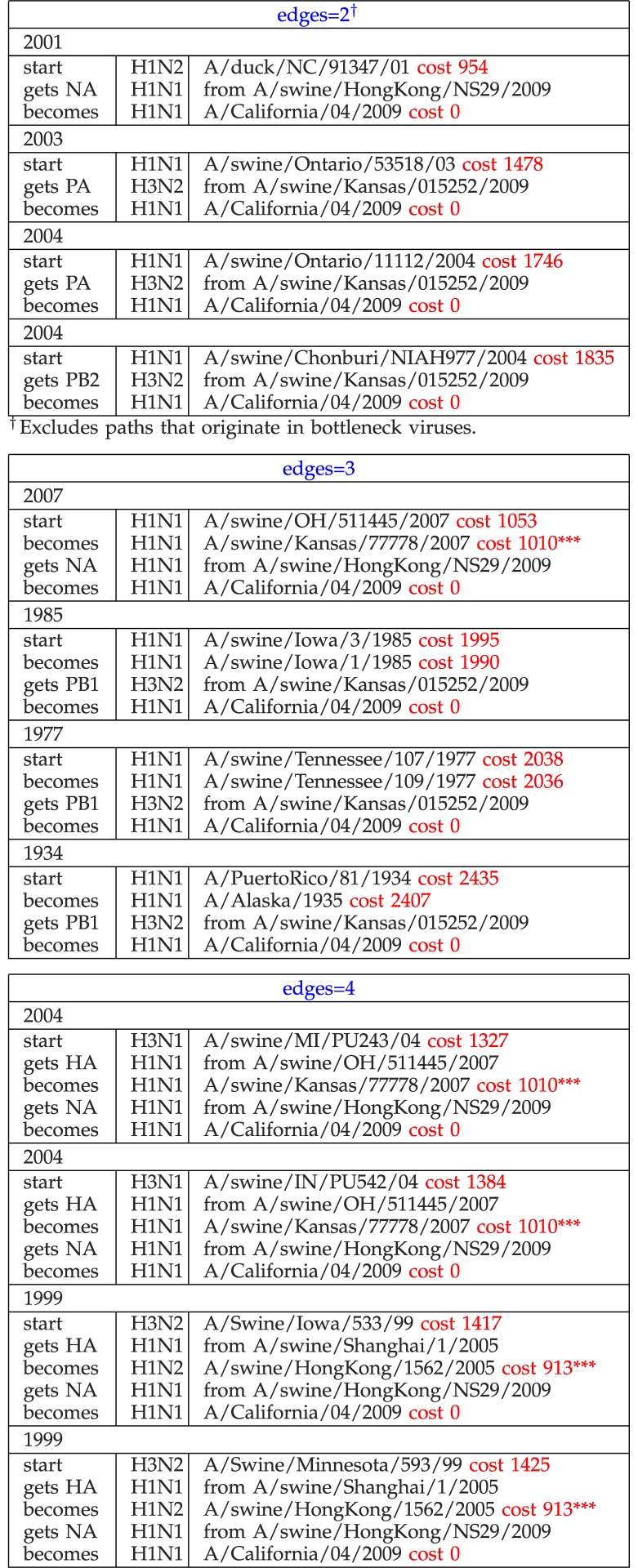
The Four Shortest Paths with Two, Three, and Four Edges Each (*** = Bottleneck) (All Reassortments Match Those in Figs. 3, 4, and 5)

**TABLE 5:** Shortest Paths with 5—10 Edges (*** = Bottleneck) (All Reassortments Except Those Noted Match Figs. 3, 4, and 5)

edges=5		
2002		
start	H5N2	A/mallard/Maryland/789/2002 cost 2667
becomes	H5N2	A/mallard/MD/790/2002 cost 2662
gets HA	H1N2	from A/swine/HongKong/1110/2006^1^
becomes	H1N2	A/swine/Guangxi/13/2006 cost 940^***^
gets NA	H1N1	from A/swine/HongKong/NS29/2009
becomes	H1N1	A/California/04/2009 cost 0
edges=6		
1998		
start	H3N2	A/Swine/Nebraska/209/98 cost 1441
gets NP	H3N2	from A/Swine/Minnesota/593/99
becomes	H3N2	A/Swine/Iowa/533/99 cost 1417
gets HA	H1N1	from A/swine/Shanghai/1/2005
becomes	H1N2	A/swine/HongKong/1562/2005 cost 913^***^
gets NA	H1N1	from A/swine/HongKong/NS29/2009
becomes	H1N1	A/California/04/2009 cost 0
edges=7		
1995		
start	H3N2	A/NewYork/687/1995 cost 2643
becomes	H3N2	A/NewYork/678/1995 cost 2637
gets PA	H3N2	from A/Swine/Minnesota/593/99
becomes	H3N2	A/Swine/Iowa/533/99 cost 1417
gets HA	H1N1	from A/swine/Shanghai/1/2005
becomes	H1N2	A/swine/HongKong/1562/2005 cost 913^***^
gets NA	H1N1	from A/swine/HongKong/NS29/2009
becomes	H1N1	A/California/04/2009 cost 0
edges=8		
1995		
start	H3N2	A/NewYork/623/1995 cost 2656
becomes	H3N2	A/NewYork/612/1995 cost 2652
becomes	H3N2	A/NewYork/635/1996 cost 2647
gets PA	H3N2	from A/Swine/Minnesota/593/99
becomes	H3N2	A/Swine/Iowa/533/99 cost 1417
gets HA	H1N1	from A/swine/Shanghai/1/2005
becomes	H1N2	A/swine/HongKong/1562/2005 cost 913^***^
NA	H1N1	from
becomes	H1N1	A/California/04/2009 cost 0
edges=9		
2003		
start	H3N2	A/NewYork/213/2003 cost 2948
becomes	H3N2	A/NewYork/215/2003 cost 2945
gets PA	H3N1	from A/swine/MI/PU243/04^3^
becomes	H3N2	A/turkey/Ohio/313053/2004 cost 1545
NA	H3N1	from A/swine/IN/PU542/04^3^
becomes	H3N1	A/swine/MI/PU243/04 cost 1327
gets HA	H1N1	from A/swine/OH/511445/2007
becomes	H1N1	A/swine/Kansas/77778/2007 cost 1010^***^
gets NA	H1N1	from A/swine/HongKong/NS29/2009
becomes	H1N1	A/California/04/2009 cost 0
edges=10		
1977		
start	H1N6	A/mallard/Alberta/42/1977 cost 3382
gets HA	H4N6	from A/gadwallduck/ALB/53/1977^3^
becomes	H4N6	A/canvasbackduck/ALB/274/1977 cost 3336
gets NS	H1N1	from A/mallard/Alberta/46/1977^3^
becomes	H4N6	A/bluewingedteal/ALB/243/1977 cost 3270
gets NA	H1N1	from A/mallard/Alberta/127/1977^2^
becomes	H4N1	A/mallardduck/Alberta/291/1977 cost 2847
gets HA	H1N1	from A/swine/OH/511445/2007
becomes	H1N1	A/swine/Kansas/77778/2007 cost 1010^***^
gets NA	H1N1	from A/swine/HongKong/NS29/2009
becomes	H1N1	A/California/04/2009 cost 0

^1^ Matches only Olsen and Kingsford, Nagarajan & Salzberg (Fig. 3).

^2.^ Matches only Smith et al. (Fig. 5).

^3^ Does not match any of Figs. 3, 4 or 5.

In Table 5, it is immediately noticeable that nonbottleneck A/Swine/Nebraska/209/98 has path length 1, 441 (with edges $= 6$) which is significantly different from other paths that are ${\approx }2{,}550$ for edges $= 5$, 7, and 8.

Turning to Table 6, we note that while the oldest shortest path is 2, 066 for A/swine/USA/1976-MA/1931, the shortest paths for later decades ($1940,\ldots, 1960$) are not significantly different. In the 1970s, some biological event results in a large multiplicity of shortest paths of length ${\approx} 2{,}000$. In subsequent decades the shortest path lengths decrease significantly, dropping to 950-1500 in the 2000s. Once again we see A/duck/NC/91347/01 standing out because it is dated 2001, unlike the remaining viruses from this decade that are from 2004 or later.

**TABLE 6:** The Five Shortest Paths by Decade^†^

Subtype	Decade	Path length
	1930–1939	
H1N1	A/swine/USA/1976-MA/1931	2066
H1N1	A/swine/USA/1976/1931	2072
H1N1	A/swine/1931	2100
H1N1	A/swine/Ohio/23/1935	2108
H1N1	A/Phila/1935	2308
	1940–1949	
H1N1	A/swine/Jamesburg/1942	2073
H1N1	A/AA/Marton/1943	2367
H1N1	A/Bellamy/1942	2401
H1N1	A/Weiss/1943	2405
H1N1	A/Hickox/1940	2415
	1950–1959	
H1N1	A/swine/Wisconsin/1/1957	2056
H1N1	A/Albany/13/1951	2704
H1N1	A/FortWorth/1950	2764
H1N1	A/Malaya/302/1954	2824
H1N1	A/Malaysia/1954	2824
	1960–1969	
H1N1	A/swine/Wisconsin/1/1961	2023
H3N2	A/HongKong/1–8-MA21–1/1968	2894
H3N2	A/HongKong/1–8-MA21–3/1968	2911
H3N2	A/Albany/10/1968	2912
H3N2	A/Beijing/1/1968	2912
	1970–1979	
H1N1	A/swine/Minnesota/5892–7/1979	2005
H1N1	A/swine/Tennessee/10/1976	2015
H1N1	A/swine/Nebraska/123/1977	2021
H1N1	A/swine/Minnesota/27/1976	2021
H1N1	A/swine/Tennessee/17/1976	2023
	1980–1989	
H1N1	A/swine/Iowa/17672/1988	1921
H1N1	A/turkey/NC/17026/1988	1934
H1N1	A/swine/Wisconsin/1915/1988	1953
H1N1	A/Swine/Indiana/1726/1988	1958
H1N1	A/swine/Kansas/3024/1987	1972
	1990–1999	
H3N2	A/Swine/Iowa/533/99	1417
H3N2	A/Swine/Minnesota/593/99	1425
H3N2	A/Swine/Nebraska/209/98	1441
H1N1	A/turkey/IA/21089-3/1992	1910
H1N1	A/swine/Maryland/23239/1991	1911
	2000–2009 (excludes bottlenecks)	
H1N2	A/duck/NC/91347/01	954
H1N1	A/swine/OH/511445/2007	1053
H3N1	A/swine/MI/PU243/04	1327
H3N1	A/swine/IN/PU542/04	1384
H1N1	A/swine/Ontario/53518/03	1478

^†^ Only the first of any set of paths originating from same location.

### 6.1 Significance of Bottleneck Viruses

As far as we are aware, the discovery of bottleneck viruses is new to the field and has not been reported elsewhere. How do these bottleneck viruses arise and what is their significance? Reviewing Table 2, we see that

1. five of the six bottlenecks were isolated from swine (the 6th., A/Iowa/CEID23/2005, was isolated from a swine farm worker [33]),
2. all bottlenecks obtained the NA segment from A/swine/HongKong/NS29/2009,
3. the NA segments of the bottlenecks are very distant from the target(A/California/04/2009)s NA, (from 363 to 1, 076 units), and
4. the donor(A/swine/HongKong/NS29/2009)s NA is only 160 units from the target’s NA.

We can, therefore, argue that there are six potential immediate sources from which the target SOIV could have emerged. A/swine/Shanghai/1/2007 and A/swine/Guangxi/13/2006(H1N2) are discussed by Yu et al. [34]. All four Chinese bottleneck viruses are included in the Table entitled “Recent ancestral swine influenza A viruses of pandemic (H1N1) 2009 viruses,” in [35], Supplementary Material]. Interestingly, this Table also includes A/swine/OH/511445/2007, which is not a bottleneck, but appears as a donor to the bottlenecks in Fig. 10 (see Section 6.2). A/swine/Shanghai/1/2007 is also included in Table 1 of Trifonov et al. [28]. Both Hong Kong viruses are listed in Table S3 in Smith et al. [30], ].

A/swine/Kansas/77778/2007 is a particularly virulent strain discussed by Ma et al. [36]. This strain also appears in the phylogenetic trees given by Kingsford et al. [29]. The donor A/swine/HongKong/NS29/2009 is listed by Smith et al. [30], Supplementary Information] and Vijaykrishna et al. [37], Online Supporting Material].

The bottleneck viruses are, thus, the key members of the large sets that have been identified by other researchers as being important to the SOIV evolution. Our algorithmic technique highlights these six viruses as being crucial to the SOIV pandemic. We were able to discover these viruses via search of a comprehensive database without reliance on preconceived notions of lineages to sample. It is now for biologists, virologists, and epidemiologists to apply molecular biology techniques to establish the functional reasons for the prominence of these viruses. Our research, which is of a purely algorithmic nature, will proceed in the directions given in Section 6.4.

One of the main accomplishments of our research is to provide means for identification of specific viral isolates that are likely similar to ancestors of epidemic viruses. By concentrating on actual viral isolates rather than inferred ancestors as in phylogenetics, we assure that our results are functionally plausible. In phylogenetics, the goal is to infer median states at ancestors to optimize an objective function over edit costs—there are typically no functional constraints attempted or implied (but see Wheeler [38] for a counterexample). The specificity and functional constraints inherent to our method are important because they permit other researchers, especially in molecular biological domains, to use our results to choose *in vitro* and *in vivo* models.

### 6.2 Nonbottleneck Viruses of Interest

In addition to the bottleneck viruses, A/duck/NC/91347/01, A/swine/OH/511445/2007, and A/Swine/Nebraska/209/98 are worthy of detailed study because of their unusually low distance to the target.

### 6.3 Validation of Reassortments

It is of great interest to compare the reassortments in the paths of Tables 4 and 5 against the models described in Section 3. We consider a reassortment indicated in our in-tree as matching a reassortment in Figs. 3, 4, or 5, if there is a path in the figures matching (in terms of subtype, host) the reassortment from the in-tree. For example, in Table 4 the three-edge path for 1977 gets PB1 from H3N2 swine. There is a path from H3N2 swine to SOIV in each of Figs. 3, 4, and 5.

All reassortments in Table 4 and most in Table 5 match the models of Olsen and Kingsford, Nagarjan and Salzberg (Fig. 3), Trifonov, Khiabanian, and Rabadan (Fig. 4), and Smith et al. (Fig. 5). Those that do not match are indicated by footnotes in Table 5.

As stated in Section 3, the unavailability of enumerated lists of classes of viruses (e.g., “Classical H1N1 swine,” etc.) limits the granularity of our validation. Nevertheless, we see a broad agreement between our reassortments and the results of other researchers, which indicates that our research is consistent with, and a useful addition to, the existing knowledge and methods base. The in-trees generated by our algorithm provide an alternate and functionally actionable model for analyzing the evolution of S-OIV and other viruses.

### 6.4 Future Research

Some issues that present themselves for future work are:

1. Bokhari and Janies [19] have proposed means for incorporating temporal, geographic, and host constraints in reassortment networks. These would result in more precise analyses.
2. Given that fine grained data are available for the present pandemic, it is of great interest to monitor *intra*-SOIV evolution, and
3. Refine the algorithm to repeat this analysis at a finer granularity, using distances between specific proteins encoded in segments, rather than global distances between segments.
